# Supplementary material for: Inhibition of mTORC1 by lncRNA H19 via disrupting 4E-BP1/Raptor interaction in pituitary tumours
Source: Nat Commun. 2018 Nov 5;9:4624. doi: 10.1038/s41467-018-06853-3 (PMC6218470; doi:10.1038/s41467-018-06853-3)
Supplement: Supplementary file 1 — Supplementary Information [file 41467_2018_6853_MOESM1_ESM.pdf]

Inhibition of mTORC1 by lncRNA H19 via disrupting 4E-BP1/ Raptor interaction in pituitary tumours

Wu et al.

Supplementary Figure 1

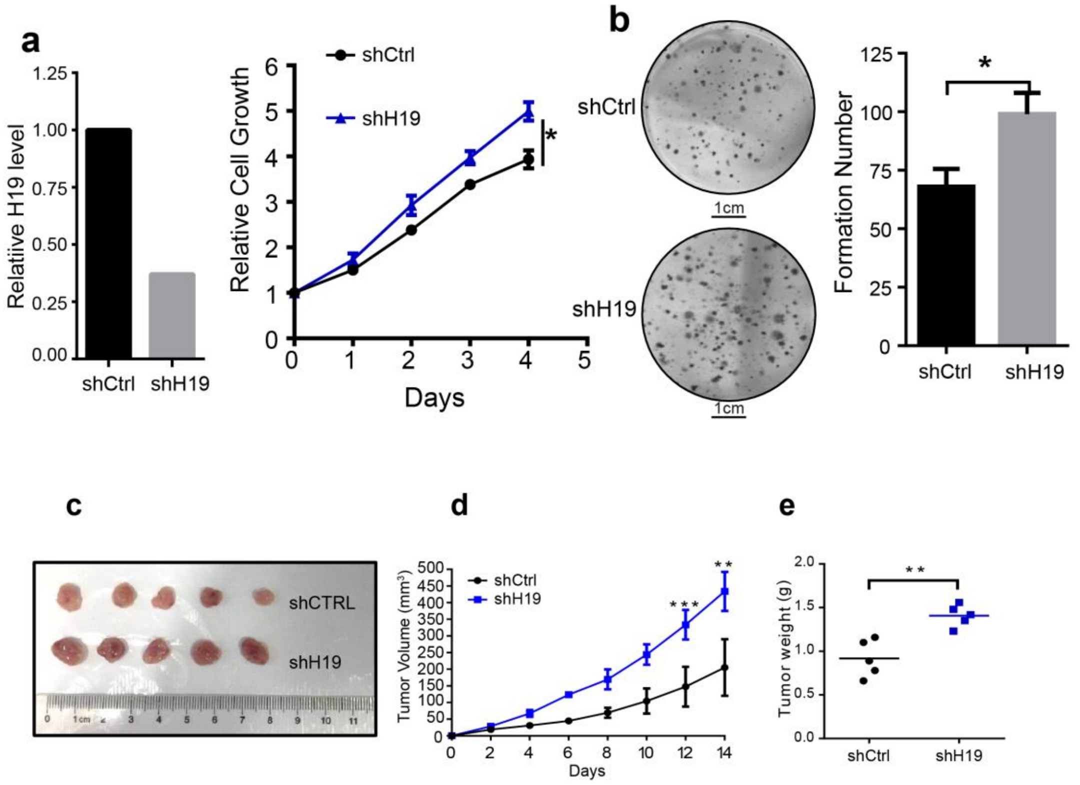

Supplementary Figure 1 | H19 knockdown enhances GH3 cell proliferation. (a) GH3 cells were infected with lentiviral H19 shRNA or a control shRNA. H19 RNA levels were measured by qRT-PCR and normalized to GAPDH. **(b)** H19 knockdown enhances GH3 cell proliferation. The proliferation of GH3 cells infected with lentiviral H19 shRNA or a control shRNA was periodically analysed with an MTS assay (**p*<0.05). **(c)** H19 knockdown enhances the colony formation rate of GH3 cells. Cells stably expressing shH19 or a control shRNA were seeded into 6-well plates with 200 cells per well and cultured for 10 days, followed by crystal violet staining and colony counting (scale bar, 1cm. **p*<0.05). **(d-f)** H19 knockdown accelerates GH3 tumour growth *in vivo*. H19 stably knocking down GH3 cells (1×10^6) (shH19 group) or control GH3 cells (1×10^6) (shCTRL group) were subcutaneously grafted into nude mice. At the end of the experiments, mice bearing tumours were sacrificed, and the tumours were collected, photographed **(d)** and weighed **(f)**. **(e)** The growth of xenograft tumours was measured by tumour volume every other day (volume=width^2×length×1/2). Error bars are the mean ± SEM values.

Supplementary Figure 2

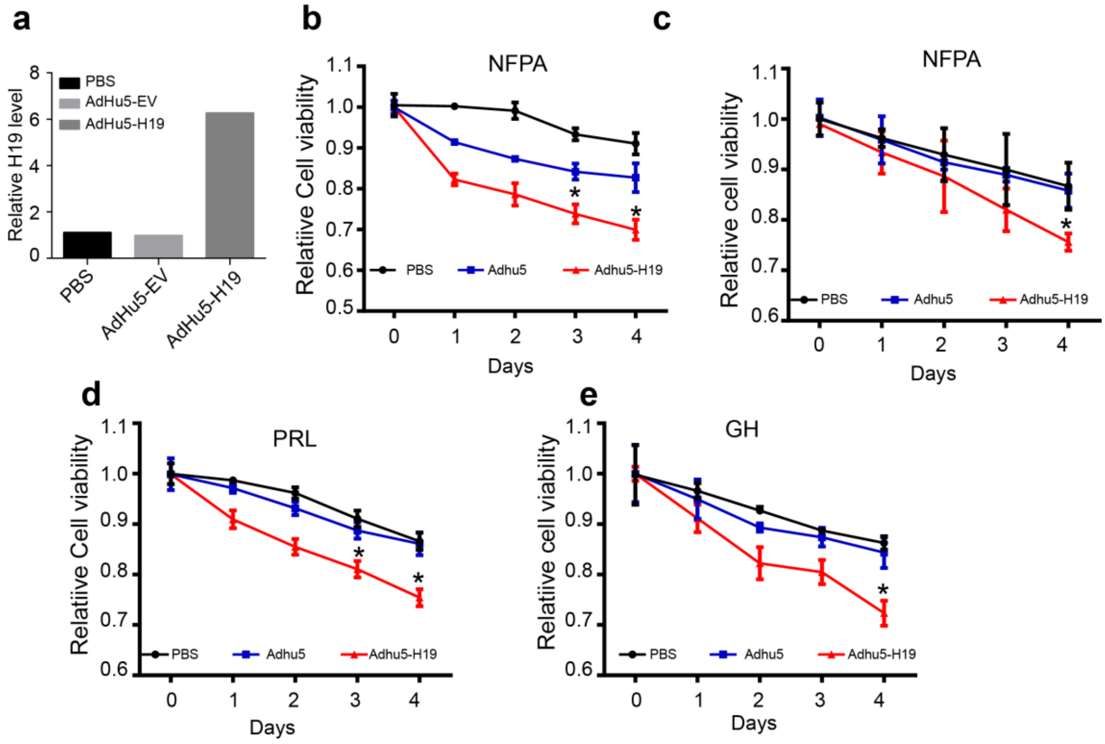

Supplementary Figure 2 | H19 suppresses human primary pituitary tumor cell growth.

(a) Human primary pituitary tumor cells were infected with adenovirus harboring empty vector or H19 expressing vector (MOI=10), 48 hours late, the total RNA was prepared and the expression of H19 was detected by qRT-PCR. **(b-e)** The viability of 1 GH tumour, 1 PRL tumour, and 2 NFPA tumour primary cells infected with H19 adenovirus **(**MOI=10) or control adenovirus was periodically analysed with MTS assays for 4 days (Error bars are the mean ± SEM values, **p*<0.05; n=5).

Supplementary Figure 3

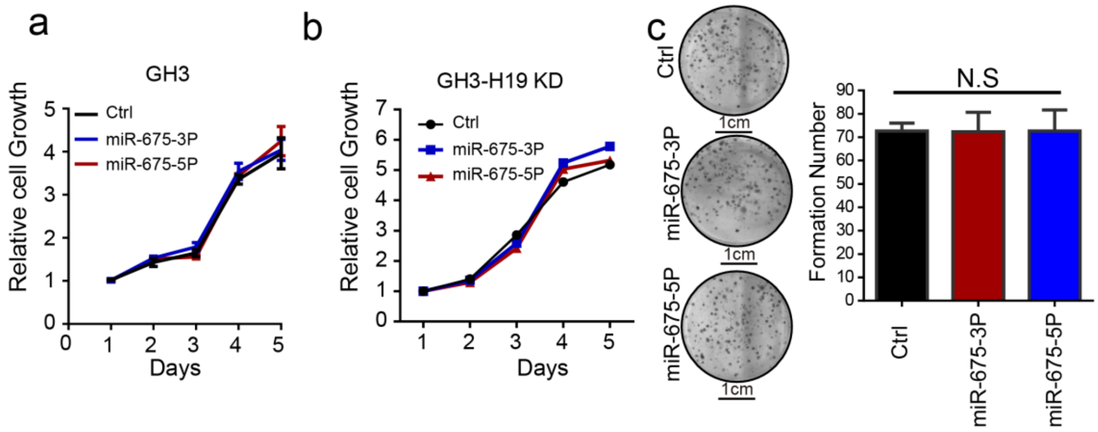

Supplementary Figure 3 | H19 inhibits GH3 cell growth independent of miR-675.

(a-b) GH3 and H19 KD GH3 cells were transfected with miR-675-3p and miR-675-5p, and the proliferation was periodically analysed with an MTS assay. **(c)** GH3 cells were transfected with miR-675-3p and miR-675-5p, a colony formation assay was performed. Error bars are the mean ± SEM values. scale bar, 1cm.

Supplementary Figure 4

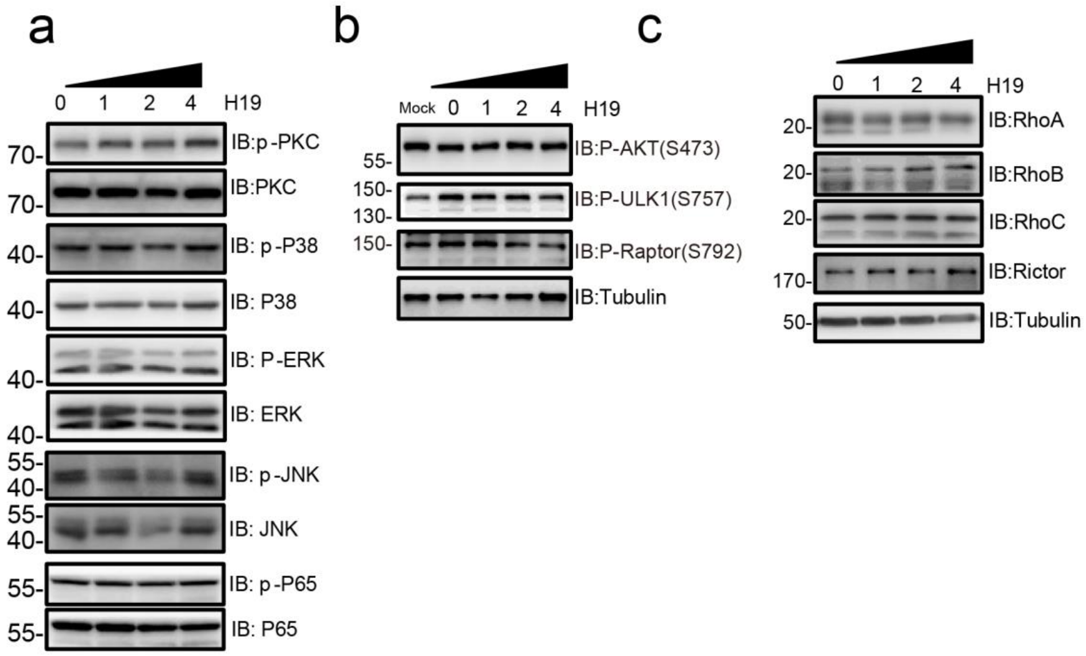

Supplemental Figure 4 | H19 has no effect on PKC/ERK/p38, JNK/p65 pathways and mTORC2 activity. (a) H19 overexpression has little effect on major pathways related to tumourigenesis. Whole-cell lysates derived from H19-transfected GH3 cells were collected at 36 h post-transfection. The expression of ERK, p-ERK, p38, p-p38, JNK, p-JNK, p-65, and p-p65 in GH3 cells was analysed by immunoblotting. **(b)** H19 does not change the mTORC2 activity in GH3 cells transfected with increasing doses of H19. At 48 h post-transfection, the cells were harvested, and the levels of p-AKT (S473), p-Ulk1 (S757), and p-Raptor (S792) were analysed by immunoblotting. **(c)** H19 has no effect on cytoskeletal remodelling in GH3 cells transfected with increasing doses of H19. At 36 h post-transfection, the cells were harvested and the RhoA, RhoB, RhoC and Rictor expression levels were analysed by immunoblotting.

Supplementary Figure 5

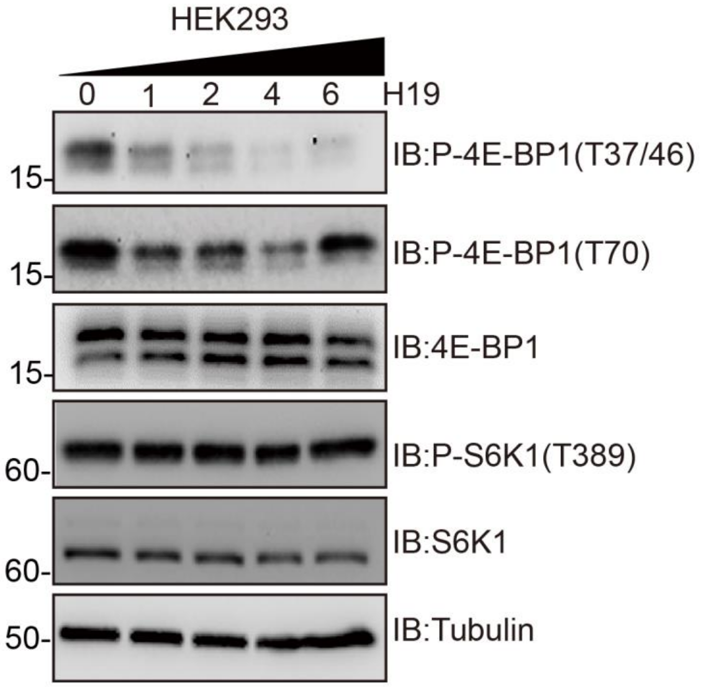

Supplemental Figure 5 | H19 overexpression suppresses 4E-BP1 phosphorylation in HEK293 cells. After 48 h post-transfection with increased doses of H19, whole-cell lysates were prepared, and the levels of phosphorylated 4E-BP1 Thr70, phosphorylated 4E-BP1 Thr37/46, total 4E-BP1, total S6K1, and phosphorylated S6K1 were examined by western blotting with the indicated antibodies. Tubulin was used as the loading control.

Supplementary Figure 6

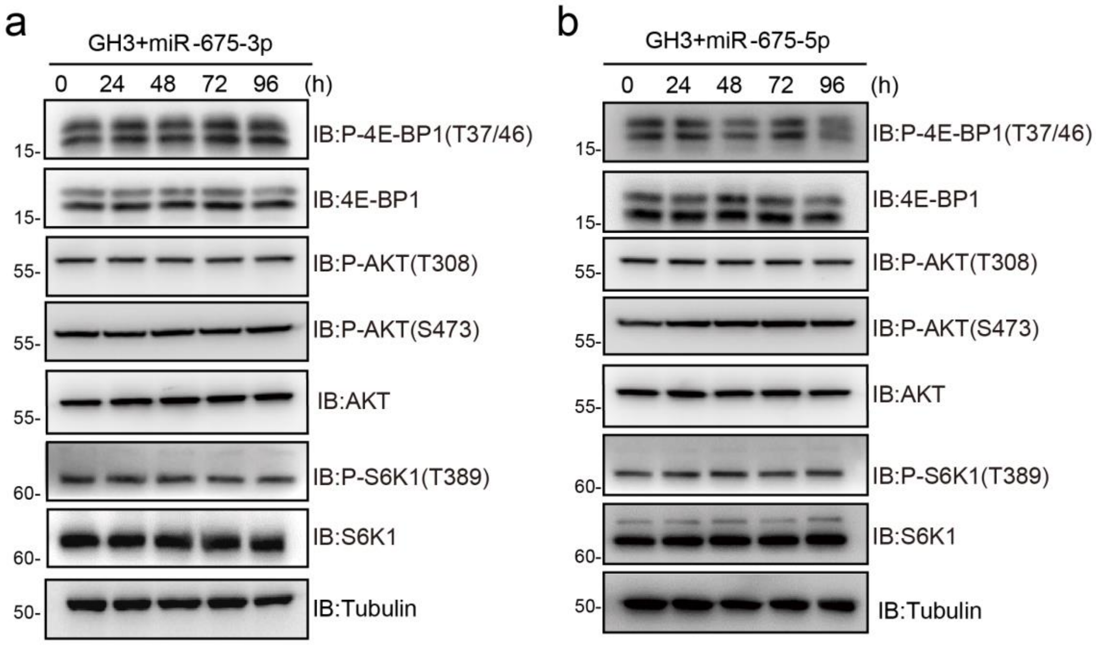

Supplementary Figure 6 | miR-675 had no effect on 4E-BP1 phosphorylation in GH3 cells.

(a-b) Western blot results showing that after time-dependent treatment with mir-675-3p or miR-675-5p, the phosphorylation of 4E-BP1 and S6K1 had not changed.

Supplementary Figure 7

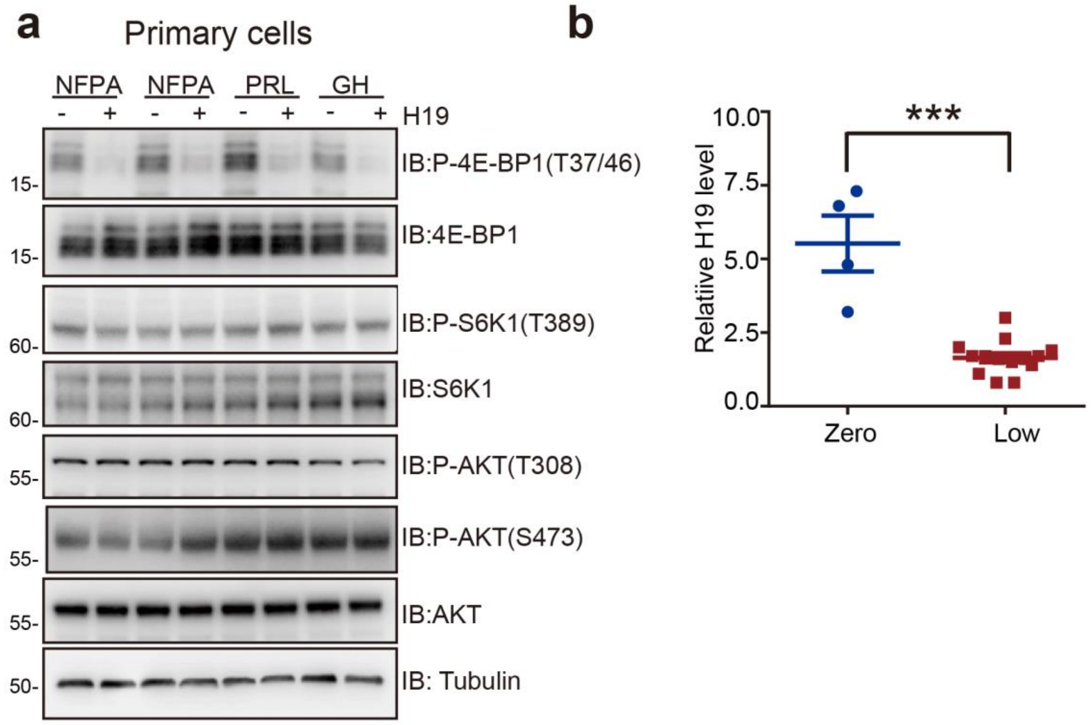

Supplementary Figure 7 | H19 suppresses 4E-BP1 phosphorylation in primary human pituitary tumor cells. (a) Human primary pituitary cells were infected with adenovirus harboring empty vector or H19 expression vector (MOI=10). 48 hours post infection, the whole-cell lysates were subjected to SDS-PAGE and immunoblotted with the indicated antibodies. Tubulin was used as the loading control. **(b)** 4E-BP1 phosphorylation level and H19 expression level were analysed in 18 human primary pituitary tumour tissues via immunohistochemical (IHC) staining and qRT-PCR, respectively. The tissues were categorized into two groups according to the expression level of p-4E-BP1 and the relationship between the p-4E-BP1 score and H19 expression level was analysed, Error bars are the mean ± SEM values, ***<0.001.

Supplementary Figure 8

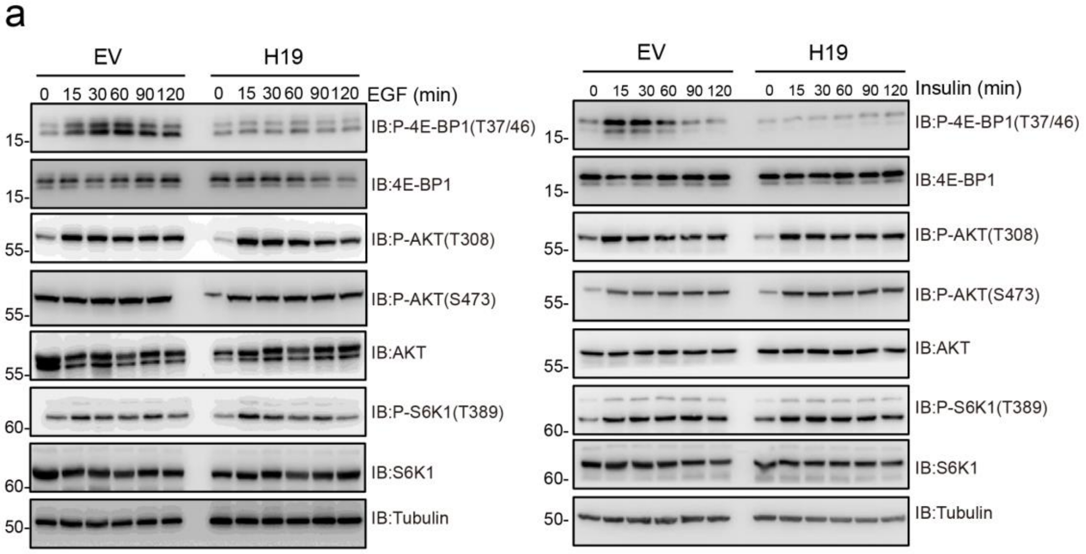

Supplemental Figure 8 | H19 attenuates mitogen-induced 4E-BP1 phosphorylation. (a) H19 inhibits the ability of insulin to stimulate 4E-BP1 phosphorylation. GH3 cells expressing H19 vector or EV were serum starved for 24 h and then collected after insulin (100 ng ml^{-1}) stimulation for the indicated period of time. Whole-cell lysates were subjected to immunoblot analysis with the indicated antibodies. **(b)** H19 inhibits the ability of EGF to stimulate 4E-BP1 phosphorylation. Same as **(a)**, except EGF (100 ng ml^{-1}) was used.

Supplementary Figure 9

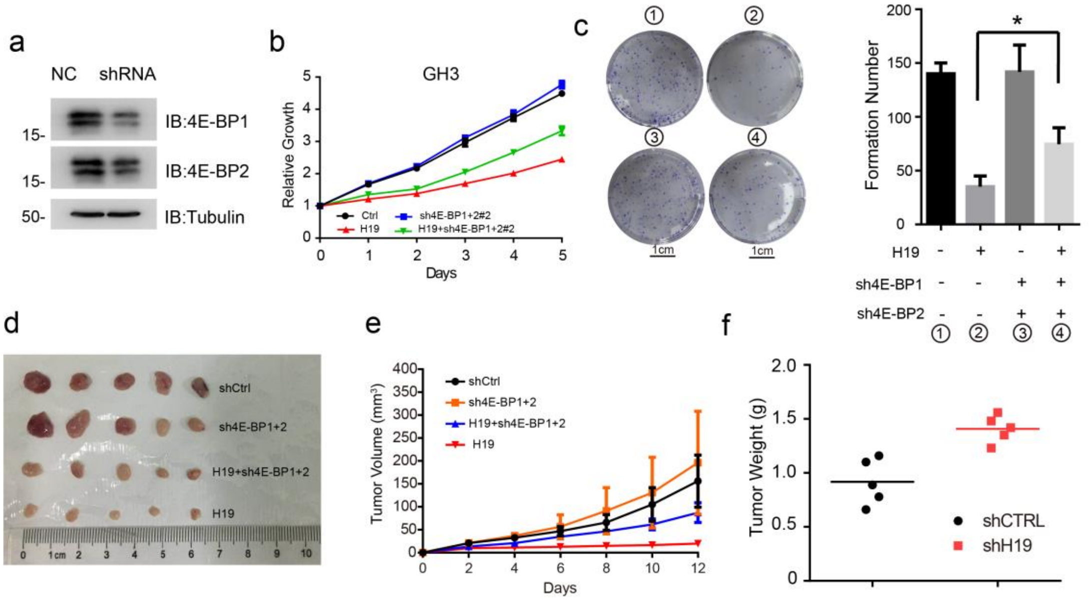

Supplementary Figure 9 | H19 suppresses pituitary tumour proliferation through the H19-mTORC1-4E-BP1 axis. (a) GH3 cells were simultaneously infected with lentiviral shRNAs for both 4E-BP1 and 4E-BP2, or for a control shRNA. 4E-BP1 and 4E-BP2 protein levels were monitored by immunoblotting. **(b)** 4E-BP1 and 4E-BP2 double knockdown rescues the H19-mediated GH3 cell growth suppression. Cell viability was detected daily for 5 days using MTS assays and is expressed as relative proliferation (fold change over value on day 1). Error bars represent the SDs in triplicate. **(c)** A colony formation assay shows that 4E-BP1 and 4E-BP2 double knockdown rescues the H19-mediated GH3 cell growth suppression. Cells were seeded into 6-well plates with 200 cells per well and cultured for 10 days, followed by crystal violet staining and colony counting. scale bar, 1cm. (**d-f**) 4E-BP1 and 4E-BP2 double knockdown rescues the growth of H19-overexpressing GH3 xenograft tumours. After mice bearing tumours were sacrificed, the tumours were collected, photographed **(d)** and the tumour weight was measured in each group at the end of the experiment (**f**). The data are presented as the mean±SEM. The growth of GH3 xenograft tumours was measured by tumour volume. Tumour size was monitored every two days **(e)**. The data (mean±SD; n=5) were analysed using a sample-paired Student's *t*-test.

Supplementary Figure 10

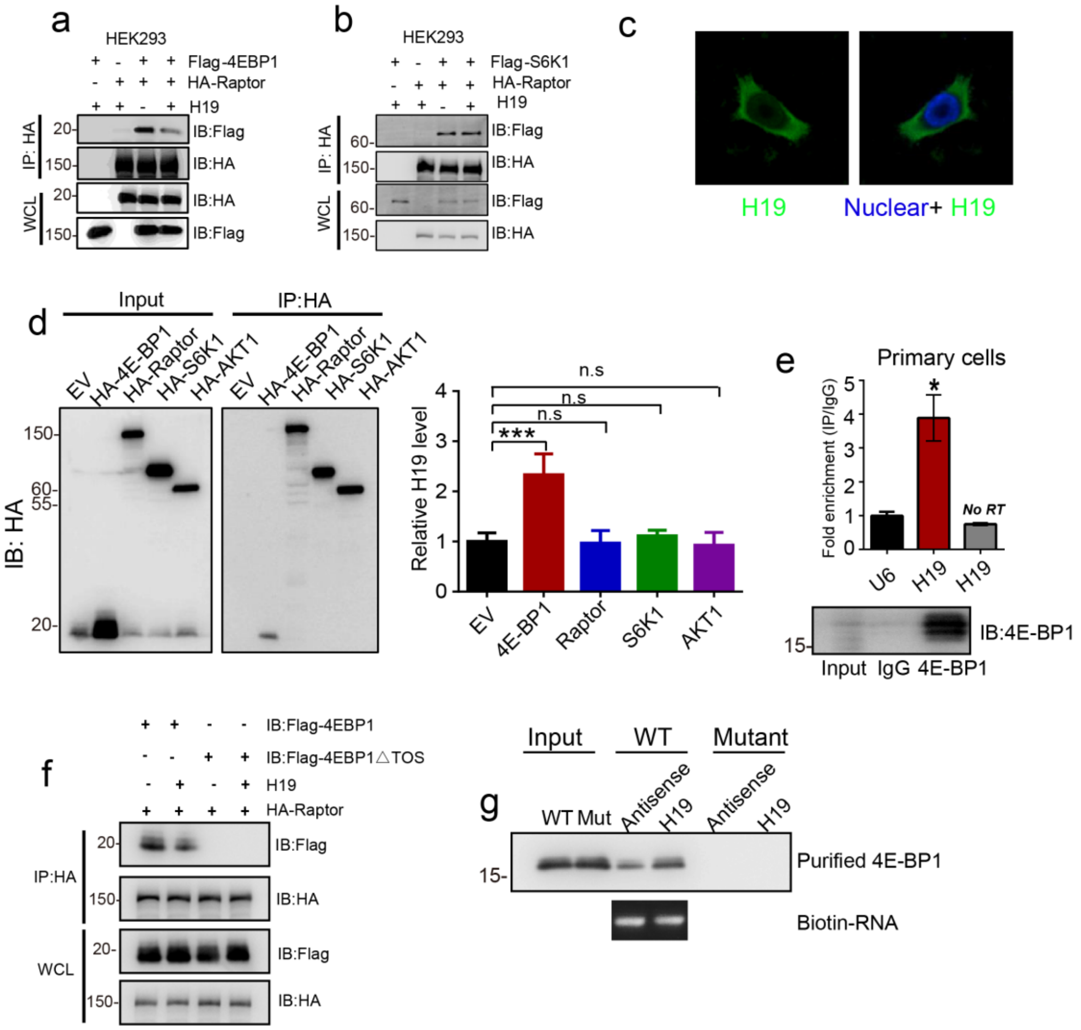

Supplementary Figure 10 | H19 disrupts 4E-BP1 binding to Raptor. (a) H19 disrupts 4E-BP1 binding to Raptor in HEK293 cells. HA-tagged Raptor and Flag-tagged 4E-BP1 were expressed in HEK293 cells with or without H19. The amount of Flag-4E-BP1 bound to HA-Raptor was analysed via immunoblotting. **(b)** H19 has no effect on Raptor and S6K1 interaction. HA-tagged Raptor and Flag-tagged S6K1 were expressed in HEK293 cells in the presence or absence of H19 overexpression. The amount of Flag-tagged S6K1 coimmunopurified with HA-Raptor was analysed via immunoblotting. **(c)** Confocal FISH image showing cytoplasmic localization of H19 in GH3 cells. **(d)** H19 does not associate with Raptor, S6K1 or AKT in GH3 cells. Whole-cell lysates of GH3 cells were immunoprecipitated with anti-HA antibody followed by transfection with HA-tagged vectors

for Raptor, S6K1, AKT, and 4E-PB1 and EVs. Aliquots of whole-cell lysates (10% of input) and the HA immunoprecipitation were resolved by SDS-PAGE, and the specific immunoprecipitation of HA was analysed via immunoblotting. The immunoprecipitation was analysed for the presence of H19 via qRT-PCR. Signals were normalized to U6 mRNA levels. The results are presented as the mean±SD of three independent experiments. **(e)** H19 interacts with 4E-BP1 in primary cells. Whole-cell lysates of primary cells were immunoprecipitated with anti-4E-BP1 antibody or control IgG. Aliquots of whole-cell lysates (1% of input) and the 4E-BP1 immunoprecipitation were resolved by SDS-PAGE, and the specific immunoprecipitation of 4E-BP1 was analysed by immunoblotting. The immunoprecipitation was analysed for the presence of H19 via qRT-PCR. ***p<0.001. **(f)** 4E-BP1 TOS motif is required for the binding of 4E-BP1 to Raptor. HA-tagged Raptor and Flag-tagged 4E-BP1 or Flag-tagged 4E-BP1 TOS deletion mutant were co-expressed in GH3 cells. The amount of Flag-4E-BP1 and Flag-4E-BP1 TOS deletion mutant bound to HA-Raptor were analysed via immunoblotting. **(g)** Purified full-length 4E-BP1, but not TOS domain-deleted 4E-BP1 protein, associates with H19. H19 transcripts and H19 antisense were labelled with biotin and incubated with incubated purified wild type 4E-BP1 or TOS domain-deleted 4E-BP1 protein. The capacity of 4E-BP1 proteins binding to H19 was analysed by immunoblotting.

Supplementary Figure 11

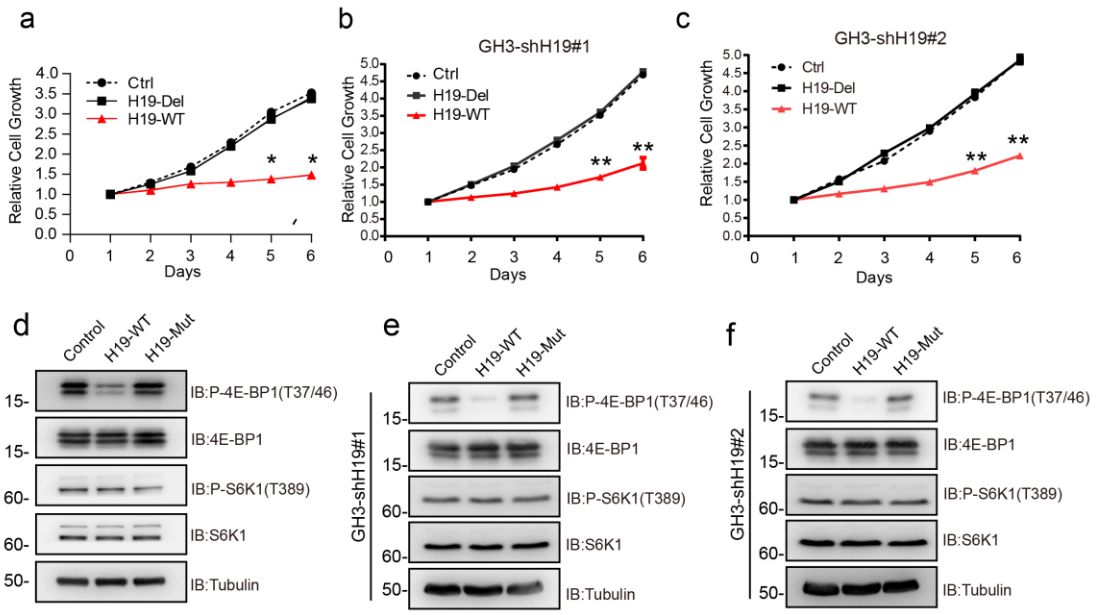

Supplementary Figure 11 | The 4E-BP1-binding-deficient H19 mutant has no effect on GH3 cell proliferation or 4E-BP1 phosphorylation. **(a)** GH3 cells were transfected with full length H19 or 4E-BP1-binding-deficient H19 mutant constructs and cell viability was periodically measured with MTS assays for 6 days. **(b-c)** GH3 cells with H19 stably knockdown were transfected with full length H19 or 4E-BP1-binding-deficient H19 mutant constructs and cell viability was measured daily with MTS assays for 6 days. **(d-f)** Normal GH3 cells and H19 knockdown GH3 cells were transfected with wilt type H19 or 4E-BP1-binding-deficient H19 mutant vectors, fourthy-eight hours post transfection, whole-cell lysates were prepared, and the levels of phosphorylated 4E-BP1, total 4E-BP1, phosphorylated and total S6K1 were examined by immunoblotting with the indicated antibodies. Tubulin was used as a loading control.

Fig.3c

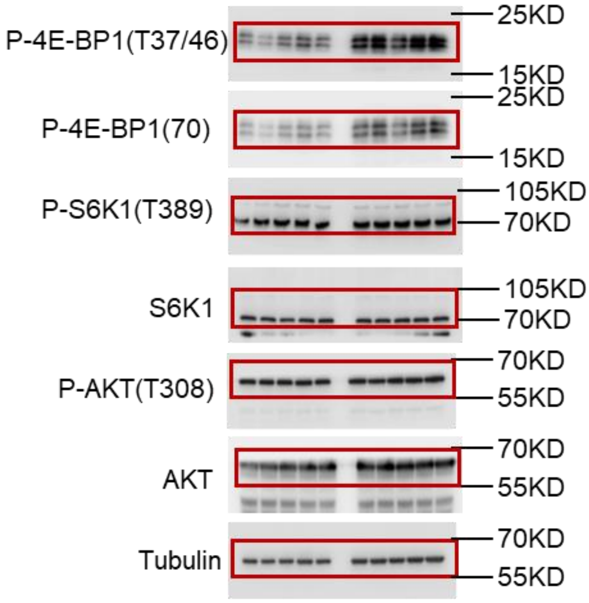

Fig.3e

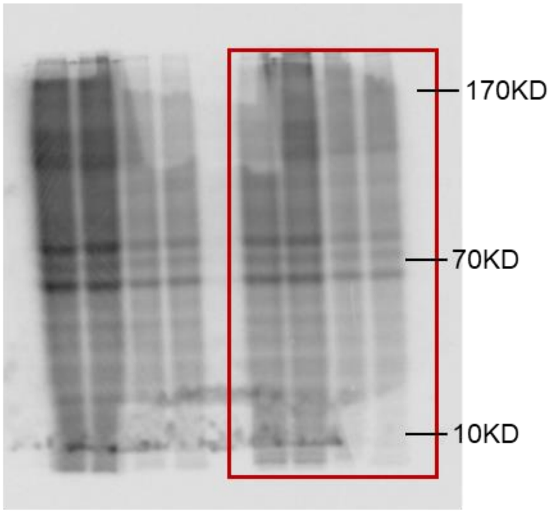

Fig.5a

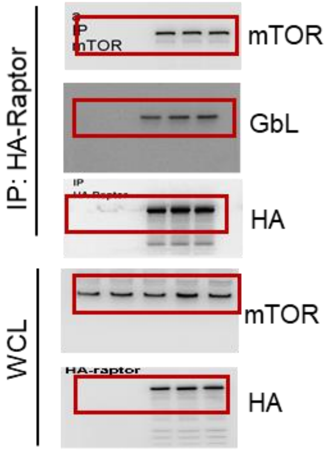

Fig.5b

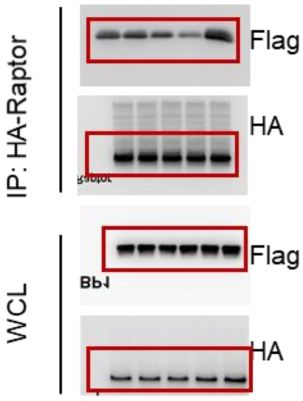

Fig.5c

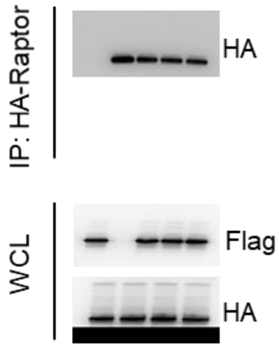

Fig.5d

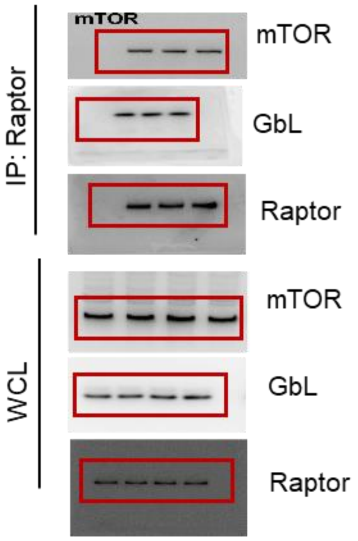

Fig.5e

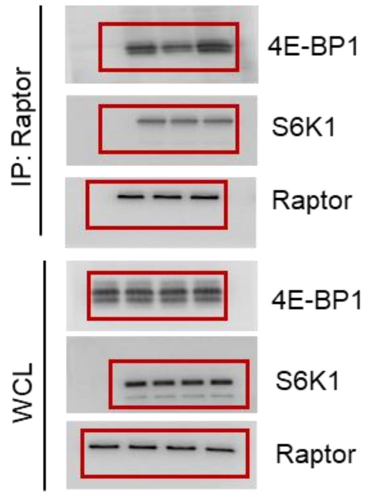

Fig.5h

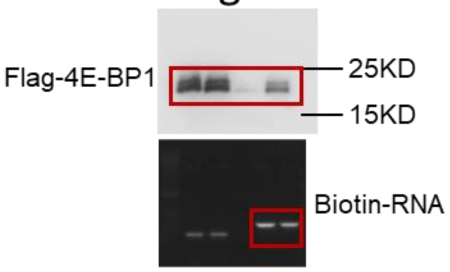

Fig.5i

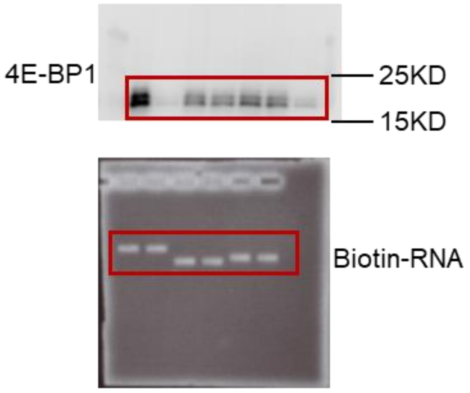

Fig.5j

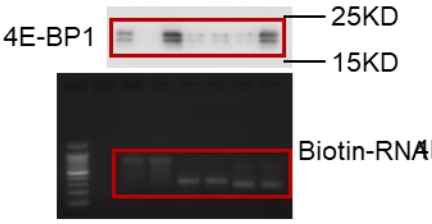

Fig.5k

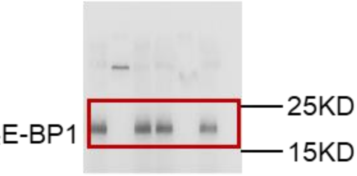

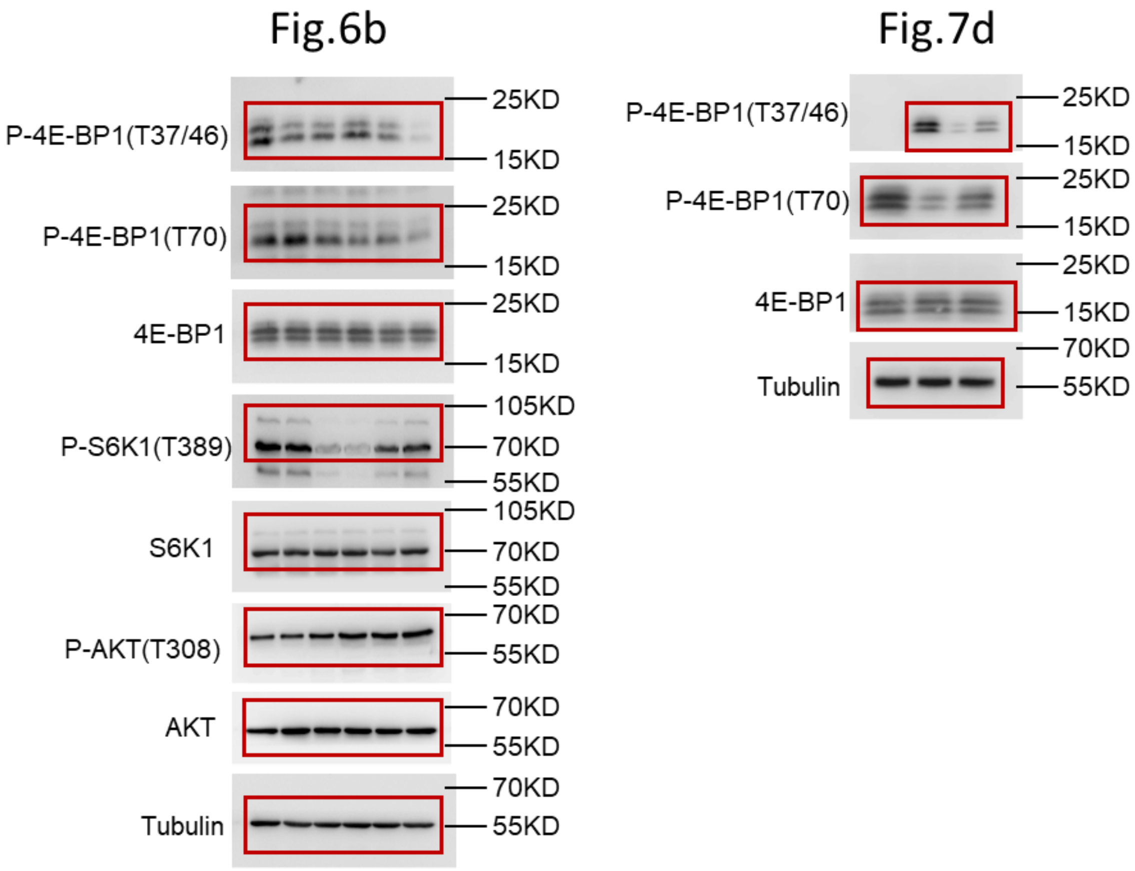

Supplementary Figure 12. Part of the uncropped scans of the western blots.

Supplementary Table 1: lncRNA expression profile between normal pituitary glands and prolactinoma tissues.

Gene Symbol	normal pituitary glands				prolactinoma tissues				
MIR503HG	14.710938	14.894001	14.399596	14.902264	6.7508437	8.6420886	7.8241287	7.5880884	7.1345311
LOC286186	9.1796624	9.2117611	10.914439	10.604968	5.3256727	5.3990927	5.9746488	4.9459217	5.523882
SOX9-AS1	8.480579	7.3675039	7.0950101	7.8929842	3.833923	2.8254972	4.5685768	4.158172	4.077688
LOC100130899	7.2571894	7.7977277	6.6645924	7.5694585	2.9326931	4.551375	3.3234002	3.7758625	3.9168547
LOC100288911	9.267725	8.7629349	8.3277388	8.4878811	4.9734486	5.6727141	4.7520906	4.8983393	5.7162421
LINC00548	10.413556	9.9617358	10.757425	9.5735482	6.7113773	6.3811788	7.128744	6.2391967	7.0355987
MIR31HG	6.6381312	7.1511201	7.2981561	8.0255422	4.1543792	4.0534552	4.1378279	4.2102491	4.1378279
KCNIP4-IT1	8.4543413	9.6626865	6.2059927	7.6356788	4.718236	5.0393669	4.7105079	4.5163434	5.3457674
LOC283070	10.275973	10.422962	9.4692492	9.8989238	6.9317815	6.2913587	7.0200516	6.553786	7.7575622
LINC01102	6.7904815	7.7634876	7.4334098	7.1417325	4.5104213	4.6659161	4.4713229	3.4810996	3.9168547
PROSER2-AS1	7.1438062	7.05098	7.0835879	7.1514373	4.718236	5.950794	4.5685768	3.1309681	3.6305461
LOC115110	6.785586	7.3493715	8.1901837	8.6666486	5.430794	5.5880837	4.8397181	5.2393916	5.4861579
TTTY13	5.6405402	5.5843299	6.3719545	5.6378263	9.6466896	6.9227058	7.3996322	8.1510683	8.7731729
LINC00478	8.7818542	8.7200654	7.5369336	8.1609471	6.4995104	6.5883992	5.4587415	4.718236	6.4053152
LINC00908	7.87293	7.5539476	7.2098574	7.5529876	4.340577	8.1518611	4.1378279	4.8983393	4.424852
LINC00599	5.1386899	5.4266072	5.9732142	5.341326	9.2901144	8.8889645	6.3549803	6.9446486	7.4515528
LOC400456	7.2605746	7.752867	8.0966866	7.6797718	4.6534302	5.0638354	6.0942932	4.8983393	6.3483556
LINC00880	5.69812	4.8968268	7.5837472	6.0710109	4.1275472	4.0534552	3.898833	3.1309681	3.9168547
KRTAP5-AS1	3.2471052	3.2683163	3.3894234	3.708486	5.8019723	5.4339261	5.3446583	5.6039292	5.4494857
LOC729970	8.1229617	8.2741345	7.4742958	7.9352252	5.9198515	7.899985	5.8076179	4.5818156	5.2122856
LINC00242	5.9317175	7.1452401	7.2698065	6.9293792	3.752637	5.5880837	4.6134702	4.718236	5.4076147
HCG4	9.2796705	9.4708702	10.19138	10.030078	8.2523441	8.0607511	7.0597928	7.4715042	8.0413728
LOC375196	8.0507545	8.6033165	9.2019192	9.3393856	7.360388	7.8866162	7.3540638	6.0595653	5.6214353
DIRC3	5.9938771	6.2403898	6.0446172	6.3795552	3.833923	4.2487024	4.8846691	4.2102491	4.33835
ERICH1-AS1	6.5215154	6.512408	6.6826991	6.6088677	5.5596893	3.9522364	5.0046792	4.3692192	4.7933215
SOX2-OT	8.2888411	7.0462123	6.0239807	6.8444416	5.8621155	5.7298982	4.7520906	4.5163434	5.1877562
DKFZp779M06	8.0151235	7.8542502	8.0914067	8.4638215	6.0956124	5.950794	6.7005813	6.9684244	6.140719
FLJ23867	6.6910135	6.7003646	6.3232387	6.2296159	7.4221506	9.3456358	7.7024599	7.7977277	8.6065587
LINC00336	5.3851362	4.7113686	5.6486512	5.8433924	3.6593774	3.1914389	3.4234381	4.718236	4.1378279
FGF13-AS1	7.3694138	7.0165738	7.0835879	7.5694585	5.8836564	5.5277962	4.6605783	5.1037161	7.3595647
DLG5-AS1	9.8746129	10.080082	10.082994	9.7962083	8.3475974	8.2752263	8.5852943	8.0487979	8.8308446
MAP3K14-AS1	4.6888214	5.4669193	5.9003586	5.973573	7.0004672	6.7508437	6.9492163	6.9145072	7.345773
GRIK1-AS1	6.5339753	5.9183614	5.9138817	6.6322349	4.5780455	5.1715854	5.1141674	4.4824583	4.5044747
NR2F1-AS1	7.3694138	6.9895104	6.652095	6.8708758	5.2183533	5.2376003	5.6656261	4.9459217	6.3951735
LINC00982	8.0007618	8.3745405	6.4580613	6.6778635	5.7188125	5.5587332	6.1856972	6.0969303	6.2084335
FLJ35934	6.6525432	6.499012	5.7584953	5.8659858	7.6281782	7.2918403	8.3103751	8.1399233	6.4138007
LINC00282	5.6876614	5.4401701	5.4951821	4.868555	3.9198365	4.0534552	3.4234381	4.0037333	4.6605783
MGC16275	6.785586	7.1354934	7.1580816	7.6398667	6.3930293	5.8817462	5.7584953	5.2805697	5.8736953
LOC339975	4.7637545	6.0969303	4.8538189	4.1480358	3.8897371	3.833923	3.0018526	4.0534552	3.4484329
ARHGEF3-AS1	5.443282	5.0638354	5.4566898	5.2280089	6.8629576	6.0418827	6.5185679	6.8432642	6.7167452
ACVR2B-AS1	6.3383612	6.7126674	7.2738908	7.0584405	7.2474311	7.6346583	9.2526896	8.5064721	7.9587025
EPB41L4A-AS2	6.358556	6.512408	6.0936332	6.4303619	5.0651827	6.051431	4.6605783	4.4824583	5.2376003
LINC00641	6.2004859	6.9426342	6.6467057	6.9293792	8.234567	7.98323	7.4595153	8.0934715	7.8218391
LOC100130992	7.3902567	7.9723205	7.4919349	8.2397964	7.618231	6.2658064	6.0942932	5.952251	6.9807917
TMPO-AS1	5.6602871	5.7170994	5.7366781	5.341326	6.7113773	6.3428117	7.1069969	7.7674244	5.9938771
NIFK-AS1	5.110257	5.3234226	5.3990927	5.1135164	6.2241987	6.5446393	6.0730213	6.3405882	6.7949546
H19	5.1386899	6.2884777	5.9003586	5.2471284	4.5104213	3.9522364	5.3446583	4.5163434	4.1378279
LINC00892	5.6876614	4.8569338	5.8613402	4.9043736	4.0037333	4.551375	3.5918089	4.3692192	4.424852
LOC648691	5.0812526	4.8724081	5.0825837	5.139969	6.0956124	5.6727141	6.8420858	6.3962444	5.8613402
A2M-AS1	9.610813	9.5754404	9.3642254	9.2225554	7.8063133	9.5213751	7.9348568	8.0556767	8.2699049
LOC254099	5.0812526	5.1386899	4.8538189	4.2029236	3.3589881	4.4824583	3.5011471	3.3256504	3.8433766
LOC100268168	7.2219878	7.5229068	8.0158204	7.8965834	8.3078165	8.851	8.796022	9.2872309	8.4429858
SHANK2-AS3	8.5922032	8.3975178	8.6894331	8.7503225	9.767891	9.3745523	9.889472	9.8665461	9.4701394
C6orf100	5.110257	5.3688629	5.0825837	5.8828926	4.1543792	4.8677832	3.6664961	4.5494509	4.2861572
LOC100130417	6.9036283	6.9856024	5.9667409	7.017966	5.9551607	5.8559014	5.6182264	5.2393916	5.7162421
STAU2-AS1	7.3324077	7.218208	8.0425702	8.2397964	8.4297763	8.2904259	9.2833291	8.9619314	8.6573549
FLJ13224	4.9863066	4.8569338	4.9763158	5.4770769	6.5494606	5.7162421	6.665481	5.8973362	5.5587332

Supplementary Table 2: Patients' information of primary pituitary tumours.

Patient Number	Gender	Age	Subtype	Tumor Volume (cm^3)	H19 expression level	p-4EBP1 （T37/46） IHC	p-AKT （S473） IHC
1	F	67	normal PG		7.9		
2	F	41	normal PG		9.5		
3	M	35	normal PG		10.6		
4	F	54	PRL	9.8	3.7		
5	F	60	PRL	2.6	10.1		
6	M	35	PRL	10.8	2.0	2	2
7	M	67	PRL	22.1	1.4	1	3
8	F	32	PRL	26.3	1.6	2	2
9	F	51	PRL	5.3	1.7	1	3
10	M	42	PRL	13.7	1.6	2	3
11	F	32	PRL	3.2	1.4		
12	F	47	PRL	1.4	5.2		
13	F	45	NFPA	3.8	0.9		
14	M	26	NFPA	1.2	8.3		
15	M	65	NFPA	6.1	7.4		
16	F	44	NFPA	5.9	1.6		
17	F	65	NFPA	5.2	7.3	0	3
18	M	63	NFPA	9.7	1.3		
19	F	27	NFPA	8.0	1.6		
20	F	68	NFPA	13.2	3.0	1	2
21	M	53	NFPA	9.5	1.7	1	3
22	M	35	NFPA	7.4	6.8	0	3
23	M	45	NFPA	10.0	1.7	1	2
24	M	36	NFPA	29.3	0.8	1	3
25	F	67	NFPA	20.8	0.8	1	2
26	M	44	NFPA	10.0	4.6		
27	F	40	NFPA	4.3	4.8	0	2
28	F	51	NFPA	9.5	3.1		
29	F	67	NFPA	15.2	4.6		
30	M	47	NFPA	21.0	2.3	1	3
31	M	45	NFPA	23.2	1.5	1	3
32	F	41	NFPA	6.4	1.6		
33	M	43	GH	8.0	1.0		
34	F	40	GH	7.2	1.9	1	2
35	M	51	GH	10.0	1.7		
36	F	47	GH	3.0	3.2	0	3
37	M	51	GH	10.0	0.5		
38	M	30	GH	13.3	1.1	1	1
39	F	65	ACTH	3.2	1.4		
40	F	61	ACTH	4.5	1.3		

*M: Male; F: Female; PG: Pituitary gland; PRL: Prolactin; NFPA: Nonfunctioning pituitary adenoma; GH: Growth hormone; ACTH: Adrenocorticotropic hormone.

Supplementary Table 3: Xenograft tumor weight.

Group	Tumor weight (g)								Mean±SEM
EV	1.7	0.4	1.25	0.5	1	0.4	0.88	0.7	0.85±0.16
H19 OE	0.04	0.03	0.13	0.05	0.05	0.1	0.04	0.05	0.06±0.01
shCTRL	0.32	0.41	0.3	0.36	0.39				0.36±0.02
shH19#1	0.82	0.92	0.62	0.87	1.22				0.89±0.10
shCTRL	1.1	1.16	0.89	0.78	0.66				0.92 ± 0.09
shH19#2	1.56	1.48	1.23	1.35	1.42				1.41 ± 0.06

Supplementary Table 4: Xenograft tumor weight.

Group	Tumor weight (g)					Mean±SEM
Control	1.7	1.45	1.2	0.5	0.7	1.11±0.22
H19	0.32	0.41	0.36	0.28	0.39	0.35±0.02
H19+sh4E-BP1+2	0.82	0.92	0.62	0.87	0.42	0.73±0.09
Control	1.08	0.82	0.69	0.74	0.61	0.79±0.08
sh4E-BP1+2#2	1.23	0.88	0.76	0.7	0.68	0.85±0.10
H19+sh4E-BP1+2#2	0.45	0.4	0.42	0.38	0.39	0.41±0.01
H19	0.1	0.08	0.07	0.04	0.07	0.07±0.01

Supplementary Table 5: Rat prolactinomas volume.

Group	prolactinomas volume (mm^3)				Mean±SEM
pre-EV	82.3	70.1	67.1	61	70.1±4.45
post-EV	40.5	40.9	40.1	40.1	40.4±0.19
pre-H19	82.3	67.3	66.8	70.1	71.6±3.63
post-H19	15.8	21.2	34.6	29.1	25.18±4.16

Supplementary Table 6: Antibodies used in this study.

Antibodies	number	company	location	dilution
Tubulin	10068-1-AP	Proteintech Group	Chicago, USA	1:2000
caspase-8	ab25901	Abcam	Cambridge, MA	1:2000
S6K1	9202S	Cell Signaling Technology	Danvers, MA	1:1000
p-S6K1 Thr389	9205S	Cell Signaling Technology	Danvers, MA	1:1000
mTOR	2983	Cell Signaling Technology	Danvers, MA	1:1000
p-4EBP1 Thr70	9455S	Cell Signaling Technology	Danvers, MA	1:1000
4EBP1	9644S	Cell Signaling Technology	Danvers, MA	1:1000
p-4EBP1 Thr37/46	9459S	Cell Signaling Technology	Danvers, MA	1:1000
PARP	9542S	Cell Signaling Technology.	Dallas, TX, USA	1:1000
p-Raptor Ser792	2083	Cell Signaling Technology.	Dallas, TX, USA	1:1000
AKT	9272	Cell Signaling Technology	Chicago, USA	1:1000
p-AKT Thr308	13038	Cell Signaling Technology	Chicago, USA	1:1000
p-AKT Ser473	4060	Cell Signaling Technology	Chicago, USA	1:1000
c-caspase 3	9661	Cell Signaling Technology	Chicago, USA	1:1000
RhoA	2117	Cell Signaling Technology	Danvers, MA	1:1000
RhoB	2098	Cell Signaling Technology	Danvers, MA	1:1000
Rictor	2114	Cell Signaling Technology	Danvers, MA	1:1000
p-ULK1 Ser757	6888	Cell Signaling Technology	Danvers, MA	1:1000
ERK	9102	Cell Signaling Technology	Danvers, MA	1:1000
P-ERK	4370	Cell Signaling Technology	Danvers, MA	1:1000
P38	8690	Cell Signaling Technology	Danvers, MA	1:1000
P-P38	4511	Cell Signaling Technology	Danvers, MA	1:1000
P-JNK	4668	Cell Signaling Technology	Danvers, MA	1:1000
JNK	9252	Cell Signaling Technology	Danvers, MA	1:1000
Raptor	2280	Cell Signaling Technology	Danvers, MA	1:1000
Flag Tag	8146	Cell Signaling Technology	Danvers, MA	1:1000
HA Tag	3724	Cell Signaling Technology	Danvers, MA	1:1000
GβL	3274	Cell Signaling Technology	Danvers, MA	1:1000
Normal Rabbit IgG	2729	Cell Signaling Technology	Danvers, MA	1:1000

Supplementary Table 7: Primers used in this study.

Species	Symbol	Sequence (5'-3')
Human	H19	Forward: GACGTGACAAGCAGGACATGAC
Human	H19	Reverse: TTCCTCTAGCTTCACCTTCCAG
Human	Actin	Forward: AGCACTGTGTTGGCGTACAG
Human	Actin	Reverse: AGAGCTACGAGCTGCCTGAC
Rat	H19	Forward: TGTCAACAGGAAGGGAACGG
Rat	H19	Reverse: CAGCTGCTTTACCTCGCTCT
Rat	H19 set1	Forward: GGAAGGAGCATGGTGTGGTTCC
Rat	H19 set1	Reverse： CTACACCTTCACTGCCCAGGTC
Rat	H19 set2	Forward： CGGGATGACTTCATCATCTCCC
Rat	H19 set2	Reverse： GGGACTCCAAACCAGTGCAATCG
Mouse	HOTAIR	Forward:CAAGTCTGCAGGGGAGTCAG
Mouse	HOTAIR	Reverse:AGGACTTAGCGCTGGCATTT
Rat	Actin	Forward: ATCAAGATCATTGCTCCTCCTGAG
Rat	Actin	Reverse: CTGCTTGCTGATCCACATCTG
Rat	U6	Forward:CTCGCTTCGGCAGCACATATACT
Rat	U6	Reverse: ACGCTTCACGAATTTGCGTGTC
Rat	H19-1-100bp	Forward: GGGGTGGGGGGTAATGGGGAAA
Rat	H19-1-100bp	Reverse:AACACTCCTGCCAGACTCCAGA
Rat	H19-101-200bp	Forward: GAAGGACTGAGGGGCTAGCTCG
Rat	H19-101-200bp	Reverse:CGATGTTTTCCAGCCTCTGCAA
Rat	H19-201-300bp	Forward: GTGTGGGGTTGAAGGGCCTGAG
Rat	H19-201-300bp	Reverse:TATCCGACCAGCCGCCACGTCC
Rat	H19-301-400bp	Forward: CAGGGGAGCTGCTGGGAAGGGT
Rat	H19-301-400bp	Reverse:CTCACCTGCCCAGCAGACGGCG
Rat	H19-401-500bp	Forward: TCTCCTTCTTCTCTCTTGGCCT
Rat	H19-401-500bp	Reverse:TGTGCCATTCTGTTGGGAGGTC
Rat	H19-501-600bp	Forward: TAGAAAGGCAGGACAGTTAGCA
Rat	H19-501-600bp	Reverse:AAGCTCTTTCCACTCCGGGGTG
Rat	H19-601-700bp	Forward: TTAGAGAGAAGATAGAAGAGGT
Rat	H19-601-700bp	Reverse:ATAGTCTCTGCCACTGTCTCCA
Rat	H19-701-800bp	Forward: GGGATCCAGCAAGAGCAGAAGC
Rat	H19-701-800bp	Reverse:GGTGGGATGTGGTGGCGGCTGG
Rat	H19-801-900bp	Forward: CACCGTAATTCATTTAGAAGCA
Rat	H19-801-900bp	Reverse:TGCCCTTCTTTCCCGTTCTCCG
Rat	H19-901-1000bp	Forward: GTGCAGGGTGTCAACAGGAAGG
Rat	H19-901-1000bp	Reverse:GTGTGTCTGCAGGCTGGACCCA
Rat	H19-1001-1100bp	Forward: CATTCCCATGAGGCACTGCGGC
Rat	H19-1001-1100bp	Reverse:TGCCAGACCCAGGGACTGAGCG

Rat	H19-1101-1200bp	Forward: TGACAGACAGAACATTTCCAGG
Rat	H19-1101-1200bp	Reverse:ACCTCCCTCCCTAGAAAGCTCA
Rat	H19-1201-1300bp	Forward: TGGGTGCAGGTAGAGCGAGGTA
Rat	H19-1201-1300bp	Reverse:CCGTCGCCGCCCGCAAGGCCCT
Rat	H19-1301-1400bp	Forward: AGCAGTGATCGGTGTCTCGGAG
Rat	H19-1301-1400bp	Reverse:ACCTGTCATCCTCGCCTTCAGT
Rat	H19-1401-1500bp	Forward: GTGGTCAACGTGATAGAAAGAC
Rat	H19-1401-1500bp	Reverse:CCACGGCACCAGCCCACGGTGT
Rat	H19-1501-1600bp	Forward: GACACTGCCGTAGAAGCCGTCT
Rat	H19-1501-1600bp	Reverse:TCTTGAGGGTTCAAGGTAGGGG
Rat	H19-1601-1700bp	Forward: TGAAAGAAATGGTGCTACCCAG
Rat	H19-1601-1700bp	Reverse:GTTCTGAGTGCAGCATATTCTT
Rat	H19-1701-1800bp	Forward: CACTACACTACCTGCCTCAGGA
Rat	H19-1701-1800bp	Reverse:GGTGGGTGGGTGCTGTGTGGGT
Rat	H19-1801-1900bp	Forward: CCTGAGACTCCATCTTCATGGC
Rat	H19-1801-1900bp	Reverse:GATGAAGTCATCCCGGGCTAGA
Rat	H19-1901-2000bp	Forward: ATCTCCCTCCTGTCTTTTTTCT
Rat	H19-1901-2000bp	Reverse:AGTCACGGATGCTTTGAGTCTC
Rat	H19-2001-2100bp	Forward: CTGCTCCCCCACTCACCCCTTT
Rat	H19-2001-2100bp	Reverse:CGCTGTATACATCCATACGGAG
Rat	H19-2101-2200bp	Forward: AGTGTGTAGGCCCCTTTGGCTA
Rat	H19-2101-2200bp	Reverse:AAGGTGAGGCCTCAAGCGCACG
Rat	H19-2201-2324bp	Forward: CCCCTTGCCTAGTCTGGAAGCA
Rat	H19-2201-2324bp	Reverse:GACTGTAACTGTATTTATTGGT

Supplementary Table 8: Synthesized oligonucleotides (IDT) were cloned into pLKO.1 vector within the Age I/EcoRI sites under the human U6 promoter. The sequences of the oligonucleotides are as follows

Symbols	Number	Sequence (5'-3')
4EBP1_shRNA	sense	5' CCGGTCCGGAAATTCCTGATGGAGCTCGAGCTCCATCAGGAATTTCCGGTTTTTG
	antisense	5' TTAACAAAAACCGGAAATTCCTGATGGAGCTCGAGCTCCATCAGGAATTTCCGGA
4EBP2_shRNA	sense	5' CCGGTGCAGCTACCTCATGACTATCTCGAGATAGTCATGAGGTAGCTGCTTTTTG
	antisense	5' ACGTCGATGGAGTACTGATAGAGCTCTATCAGTACTCCATCGACGAAAAACCTAG
4EBP1_shRNA_2	sense	5'CCGGTCCGGAAGATAAGCGGGCAGCTCGAGCTGCCCGCTTATCTTCCGGTTTTTG
	antisense	5'GATCCAAAAACCGGAAGATAAGCGGGCAGCTCGAGCTGCCCGCTTATCTTCCGGA
4EBP2_shRNA_2	sense	5'CCGGTGTGTACAGAGAAGCTCCAGCTCGAGCTGGAGCTTCTCTGTACACTTTTTG
	antisense	5'GATCCAAAAAGTGTACAGAGAAGCTCCAGCTCGAGCTGGAGCTTCTCTGTACACA
H19_shRNA_1	sense	5' CCGGTCCACCGTAATTCATTTAGACTCGAGTCTAAATGAATTACGGTGGTTTTTG
	antisense	5' TTAACAAAAACCACCGTAATTCATTTAGACTCGAGTCTAAATGAATTACGGTGGA
H19_shRNA_2	sense	5' CCGGTGCAAGTGATAGGAGGCCTTCTCGAGAAGGCCTCCTATCACTTGCTTTTTG
	antisense	5' TTAACAAAAAGCAAGTGATAGGAGGCCTTCTCGAGAAGGCCTCCTATCACTTGCA
